# Antioxidant and Functional Activities of MRPs Derived from Different Sugar–Amino Acid Combinations and Reaction Conditions

**DOI:** 10.3390/antiox10111840

**Published:** 2021-11-19

**Authors:** David D. Kitts

**Affiliations:** Food Science, Food Nutrition and Health, Faculty of Land and Food Systems, The University of British Columbia, Vancouver, BC V6T 1Z4, Canada; david.kitts@ubc.ca

**Keywords:** Maillard reaction, radical scavenging, nitric oxide, transepithelial electrical resistance, tight-junction proteins

## Abstract

The Maillard reaction (MR), or non-enzymatic browning, involves reducing sugars reacting with amino acids, peptides, or proteins when heated to produce an abundance of products that contribute to sensory, nutritional, and functional qualities of the food system. One example of an important functional quality of MR relates to antioxidant capacity, which has relevance to preserve food quality and also to extend a potential role that may promote gastrointestinal health. The addition of Alphacel (10%), a non-reactive polysaccharide, to MR reactants produced small significant (*p* < 0.05) reductions in yield of soluble Maillard reaction products (MRPs), sugar loss, and color change of products formed respectively, for reducing sugars. A similar effect was also noticed for different free-radical scavenging capacity (*p* < 0.05), using chemical (e.g., 2,2-diphenyl-1-picrylhydrazyl (DPPH)), Trolox equivalent antioxidant capacity (TEAC), and oxygen radical absorbance capacity (ORAC) assays. An inflamed Caco-2 cell model revealed nitric oxide (NO) inhibitory activity for Glu-amino acid MRPs, which contrasted the NO stimulatory activity obtained with Fru-amino acid MRPs, especially when glycine was used as the amino acid. Pre-treating Caco-2 cells with Fru-glycine MRPs protected against loss in trans-epithelial resistance (TEER) (*p* < 0.05) and reduced (*p* < 0.05) disruption of Caco-2 intestinal epithelial tight-junction (TJ) protein cells when exposed to 7.5% ethanol. A low molecular weight Fru-glycine (e.g., <1 kDa) fraction contributed to the protective effect, not observed with the corresponding high molecular weight MRP fraction. The presence of Alphacel had minimal effect on generating MRPs with relative modified protection against intestinal dysfunction in cultured Caco-2 cells. Rather, different types of sugar–amino acid combinations used to generate MRPs contributed more to mitigate injury in stress-induced Caco-2 cells. With the growing evidence that MRPs have a wide range of bioactive activities, this study concludes that specificity of substrate precursors that produce MRPs in heated foods is a critical factor for antioxidant and related cellular functions that represent a healthy gut.

## 1. Introduction

Many studies which have focused on understanding the Maillard reaction (MR), also termed non-enzymatic browning, have relied on using relatively simple chemical models in aqueous media with substrates possessing carbonyl groups from reducing sugars reacting with free amino groups from amino acids, peptides, or even proteins, under controlled conditions of temperature, pH, and water activity [1,2,3,4]. As such, variables that include the type of reducing sugar (e.g., monosaccharide vs. disaccharide, pentose vs. hexose, aldehyde vs. ketose) or type of amino acid are known to influence the rate of reaction and the subsequent composition and functional capacity of Maillard end-products [2,3,5,6]. Whereas sugar type correlates with MRP reaction rates [1], amino acids such as glycine and lysine exhibit the greatest free-radical scavenging capacity [2,3]. With temperature, the duration and intensity of heating are critical factors to produce the thermal load necessary to influence the rate of MR and direction to generate specific products [7,8,9]; for example, lesser browning due to lower heating temperatures corresponds to greater production of intermediate aroma compounds generated from the reductone pathway [10]. In addition, physical factors that are germane to the food matrix can influence the mobility of reactants as well as heat transfer or phase changes, and thus impact the reaction rate which in turn can influence the composition and possibly the derived function of complex MRPs [11].

Many simplified aqueous MR model reactions have lacked a defined matrix that could potentially show the effect of influencing heat transfer kinetics on both the yield and related composition and functional activity of MR products. MRPs that exhibit antioxidant activity represents an important functional quality that is influenced by many variables, which control the quality of heated sugar–amino acid [5,12,13] and sugar–protein [14,15] products. Previous reports have identified early-stage MRPs with characteristic fluorescence character that exhibit antioxidant activity [13,16], while others reported later browning pigments, namely melanoidins, to exhibit both pro-oxidant chelating and free-radical scavenging capacity [17,18,19]. Research conducted using cell-based model systems, such as the differentiated intestinal Caco-2 cell, have confirmed MRP antioxidant activity observed in chemical assays and also indicated a possible carry-over function to include anti-inflammatory and functional properties that relate to intestinal cell viability [20,21,22]. To this end, the intercellular junctional complex, referred to as tight junctions (TJ), which maintain membrane integrity by operating as a seal between epithelial cells near the apical surface, is a vital component of the Caco-2 cell intestinal barrier. Included in this complex structure are proteins, zonula occludens (ZO) such as ZO-1, claudins that restrict paracellular diffusion of toxicants, and pathogens [23,24]. The exposure of Caco-2 intestinal cells to pro-oxidants will reduce transepithelial electrical resistance (TEER) and adversely affect TJ protein expression and localization [25,26,27,28]. Thus, the intestinal epithelial monolayer is considered a critical first line defense barrier, and therefore dietary components that protect TJ and abate potential intestinal barrier dysfunction are important to prevent systemic exposure to food toxicants and pathogens.

The purpose of this study was to generate model MRPs using different sugar–amino acid reactant types using a dry heating process. In addition, the effects of adding the presence of a nonreactive matrix, such as Alphacel, on antioxidant and other related bioactive functional capacities of derived MRPs were assessed using both chemical assays and cultured differentiated Caco-2 cells.

## 2. Materials and Methods

### 2.1. Materials

Monosaccharide and sucrose sugars, L-lysine and L-glycine, were purchased from Sigma (St. Louis, MO, USA). Alphacel Non-Nutritive Bulk, DPPH [2,2-diphenyl-1-picrylhydrazyl], Trolox, and potassium persulfate were purchased from Sigma-Aldrich (Oakville, ON, Canada) and 96-well plates from Sarstedt (Nümbrecht, Germany). ABTS [2, 2′-Azino-bis-(3-ethylbenzothiazoneline-6-sulfonic acid)] was purchased from Sigma (St. Louis, MO, USA). The ORAC assay required 2, 2-azobis (2-amidinopropane dihydrochloride (AAPH) (Wako Chemical Inc., Richmond, VA, USA), fluorescein (Sigma, St. Louis, MO, USA), and 96-well solid black polystyrene microplates (Corning™, Tewksbury MA. USA). Caco-2 cells (HTB-37) were purchased from ATCC (American Type Culture Collection. Manassas, VA., USA), as well as Minimum Essential Medium (MEM) (Sigma, St. Louis, MO, USA). Fetal bovine serum (FBS) was from Invitrogen (Burlington, ON, Canada) and antibiotics, penicillin, and streptomycin were from Sigma (St. Louis, MO, USA). Translucent 24-well inserts were obtained from BD Biosciences (San Jose, CA, USA) and TEER values were read from a Millicell^®^ ERS volt-ohmmeter (Millipore, Bedford, MA, USA). TJ primary antibodies to Claudin 4, occludin, ZO-1, and anti-glyceraldehyde 3-phosphate dehydrogenase (GAPDH) came from Abcam Inc. (Cambridge, MA, USA), with the secondary antibody from Abcam Inc. as well. A Zeiss fluorescence microscope (Carl Zeiss Microscopy GmbH, Jena, Germany) visualized cells and the Western blots were analyzed using a ChemiDoc^TM^ MP Imaging System (Bio-Rad Laboratories Ltd., Hercules, CA, USA).

### 2.2. Methods

#### 2.2.1. MRP Sample Preparation

Reducing, including xylose (Xyl), glucose (Glu), fructose (Fru), and non-reducing sugar sucrose (Suc), were mixed 1:1 (molar ratio) with glycine (Gly) or L-lysine (Lys) respectively, in the presence and absence of 10% (*w/w*) Alphacel. Alphacel, a non-reactive bulking agent, is derived from finely ground cellulose, and is easily incorporated with other formulation components. It does not contribute to browning and is stable at the temperatures used in the baking experiments. These qualities made it a useful constituent for simulating food matrices for different MR precursors. Samples were mixed well and placed in random positions on a cookie sheet in a pre-heated oven set at 150 °C for baking for 20 and 40 min, respectively. Three replicates were prepared for each sugar–amino acid mixture combination. After being baked, samples were cooled to room temperature, added to distilled water, vortexed for 5 min, and centrifuged at 3000× *g*, for 30 min at room temperature. Supernatants were filtered through Whatman filter paper (Grade 4, Sigma-Aldrich, St. Louis, USA), and the residue was re-suspended and filtered again three times to obtain a quantitative recovery of water-soluble components. Samples were then lyophilized and stored at −80 °C until analysis. Dry mixtures were stored in desiccators at 4 °C until further analysis was required. The yield of each of the different MRPs recovered from the individual sugar–amino acid mixtures was calculated from the dry weight of the soluble fraction recovered [21].

#### 2.2.2. Sugar Analysis

MRP crude samples were re-dissolved in E-Pure water and supernatants were adjusted to 50% acetonitrile (Thermo Fisher Scientific, Waltham, MA, USA) for sugar analysis, analyzed by high-performance liquid chromatography (HPLC), using an Agilent 1100 series (Santa Clara, California, USA), equipped with a Agilent, 5 µm, 4.6 × 250 mm column and a refractive index detector [21]. The mobile phase contained 75% acetonitrile and the flow rate was 1.0 mL/min. Standard calibration curves of xylose, glucose, fructose, and sucrose were built for quantitative analysis with a concentration range of 0.625–20 mg/mL. Ribose was used as an internal standard.

#### 2.2.3. Color Measurement in Soluble MRP Fraction

Color measurement was performed on a Hunter Labscan 600 spectrocolorimeter (Hunter Associates Laboratory Inc., Reston, VA, USA). The instrument was calibrated with standard black and white tiles, and parameters L* (luminosity or brightness: L* = 0 black and L* = 100 white), a* (red–green axis: redness represents positive values and greenness represents negative values), and b* (yellow–blue axis: yellowness represents positive values and blueness represents negative values) were measured. The color difference (ΔE*) between freeze-dried water-soluble samples and a reference (same samples prepared without heating treatment) was obtained using Equation (1):

(1) $$\Delta E^{*}=\sqrt{{\left(L^{*}-L_{0}^{*}\right)}^{2}+{\left(a^{*}-a_{0}^{*}\right)}^{2}+{\left(b^{*}-b_{0}^{*}\right)}^{2}}$$

where ΔE* = color difference, L*, a*, and b* are values of the processed sample, and $L_{0}^{*}$, $a_{0}^{*}$, and $b_{0}^{*}$ are values of the reference sample, respectively. Three replicates of the same weight were randomly collected from each sample and three measurements of L*, a*, and b* were carried out on each replicate.

#### 2.2.4. Free-Radical Scavenging Activity Measurements

##### DPPH Assay

The DPPH radical scavenging activity of soluble MRPs required initial dilution with distilled water and methanol [18]. Diluted MRP samples and Trolox standard solutions were incubated with 20 µL of 1 mmol/L DPPH in 100% methanol in 96-well plates, shaken for 10 min at room temperature, and absorption was measured using a spectrophotometer (Multiskan Ascent, ThermoLab systems, Helsinki, Finland) at 519 nm. The inhibition of DPPH free radical was calculated as in Equation (2):

(2) $$\%\mathrm{Inhibition}=\frac{{\mathrm{Abs}}_{\mathrm{control}}-{\mathrm{Abs}}_{\mathrm{sample}}}{{\mathrm{Abs}}_{\mathrm{control}}-{\mathrm{Abs}}_{\mathrm{blank}}}\times 100$$

where Abs_control_ = absorbance of 0.1 mmol/L DPPH alone in methanol, Abs_sample_ = absorbance of 0.1 mmol/L DPPH + MRP sample in methanol, and Abs_blank_ = absorbance of methanol solvent control in absence of DPPH or MRP.

##### TEAC Assay

The TEAC assay was performed using the ABTS radical cation (ABTS^•+^) prepared in potassium persulfate [19]. Fresh ABTS^•+^ working solution was prepared for each assay to obtain an absorbance of at least 0.4 at a 734 nm wavelength. The ABTS radical scavenging capacity of MRP samples was measured for different concentrations (0–1.0 mg/mL) and calculated as in Equation (3):

(3) $$\%\mathrm{Inhibition}=\left(1-\frac{{\mathrm{Abs}}_{\mathrm{sample}}}{{\mathrm{Abs}}_{\mathrm{control}}}\right)\times 100$$

The standard curve was linear between 0 and 25 mmol/L Trolox. Trolox equivalent antioxidant capacity (TEAC) = slope_sample_/slope_control_. Results were expressed in mmoL Trolox equivalent (TE)/g sample.

##### ORAC Assay

The ORAC assay was conducted as described previously [18,19] using 96-well solid black polystyrene microplates. Each plate was incubated at 37 °C for 15 min and 60 µL AAPH (final concentrations 12 mmol/L) was added. Fluorescence readings (Excitation wavelength = 485 nm, Emission wavelength = 527 nm) were continuously taken (0–60 min) using a microplate reader (Fluoroskan Ascent FL, Thermo Labsystems, Helsinki, Finland). Data transformation with the area under the curve was calculated to obtain the slope, as in Equations (4) and (5):

(4) $$\mathrm{AUC}=0.5+\sum_{i=2}^{59}\frac{A_{i}}{A_{1}}+0.5\times \frac{A_{60}}{A_{1}}$$

where *A*_1_ is the initial fluorescence reading and *A_i_* is the fluorescence reading at time *i*.

(5) $$\mathrm{ORAC}=\frac{{\mathrm{slope}}_{\mathrm{sample}}}{{\mathrm{slope}}_{\mathrm{Trolox}}}$$

ORAC values were expressed as µmol Trolox equivalent/g sample.

#### 2.2.5. Caco-2 Cell Culture Experiments

Caco-2 cells (HTB-37, ATCC) were cultured in complete Minimum Essential Medium (MEM) containing Earle’s salts (Sigma, St. Louis, MO, USA), supplemented with 10% FBS (Invitrogen, Burlington, ON, Canada) and 100 units/mL of penicillin and 100 µg/mL of streptomycin (Sigma, St. Louis, MO, USA). Cells were cultured at 37 °C under a 5% CO_2_ atmosphere. Cell media were changed every 2–3 days and the cells were sub-cultured weekly. Caco-2 cells from passages 21 to 37 were used for all experiments.

##### MTT Assay

Caco-2 cell cytotoxicity was assessed in all experiments using the MTT (3-(4,5-dimethylthiazol-2-yl)-2,5-diphenyltetrazolium bromide) assay to assess metabolic activity with or without MRP exposure. Specific MRP, along with a control, had 3 replications, and the control only contained medium without MRP. Cells were grown in 96-well plants and MRPs were removed after treatment and rinsed with phosphate-buffered saline (PBS), before being incubated again with 100 μL of 0.5 mg/mL MTT for 4 h in the dark at 37 °C. Sodium dodecyl sulfate (10% SDS) in HCL (0.1 M) was added to cells for 12 h to dissolve the formazan crystal. Formazan absorbance was measured at 570 nm using a Multiskan Ascent spectrophotometer (ThermoLabsystems Inc., Guelph Mills, PA, USA). Cell viability was calculated according to Equation (6):

(6) $$\mathrm{Viability}\;\left(\%\right)=\frac{{\mathrm{Abs}}_{\mathrm{sample}}}{{\mathrm{Abs}\;}_{\mathrm{negative}\;\mathrm{control}}}\times 100$$

where Abs_sample_ = absorbance of MRP sample at 570 nm, and Abs_control_ = absorbance of control in the absence of MRP at 570 nm.

##### MRP Effect on Nitric Oxide (NO) Production in Caco-2 Cells

NO production was measured in Caco-2 cells seeded in 96-well plates (Sarstedt, Nümbrecht, Germany) at a density of 10^5^ cells/cm^2^ in complete MEM (100 μL), and grown for 3 weeks prior to experiments to establish differentiation [9,21]. Caco-2 cells were incubated with different sugar–amino acid MRPs for 24 h, and then following removal of the media, were treated with 8000 U/mL INF-γ + 0.1 μg/mL PMA for 48 h. NO was determined by measuring nitrite and nitrate (NO_2_^−^ + NO_3_^−^) in the culture medium using the Griess reagent (50 µL of 1% sulfanilamide in 5% phosphoric acid and 50 µL of 0.1% *N*-(1-naphthyl)ethylenediamine dihydrochloride), after reduction of nitrate to nitrite by nitrate reductase (Sigma, St. Louis, MO, USA), with changes in color measured on a spectrophotometer (Multiskan Ascent, ThermoLabsystems, Finland) at 540 nm. Sodium nitrate was used as the standard and the percent NO inhibition/stimulation was calculated according to Equation (7):

(7) $$\mathrm{NO}\;\mathrm{Inhibition}/\mathrm{Stimulation}\;\left(\%\right)=\left(\;\frac{{\mathrm{NO}}_{\mathrm{standard}}}{{\mathrm{NO}}_{\mathrm{sample}}}-\frac{{\mathrm{NO}}_{\mathrm{standard}}}{{\mathrm{NO}}_{\mathrm{blank}}}\;\right)\times 100$$

##### Caco-2 Epithelial Monolayer Resistance (TEER) of Crude and Fractionated MRPs

Recovery of high and low molecular weight MRPs was accomplished by multistep ultrafiltration under nitrogen (40 psi) and individual fractions (LMW = <1 KDa; HMW = >1 KDa) were collected and freeze-dried. Caco-2 cells were seeded in 24-well translucent, high-density polyethylene terephthalate membrane trans-well (0.40 µm pore size, 0.3125 cm^2^ growth surface area) inserts (BD Biosciences, San Jose, CA, USA) at a concentration of 2.5 × 10^5^/cm^2^ and incubated for 21 days to establish differentiation. Monolayer integrity was checked initially using 100 µM of Lucifer yellow applied to the apical side of the insert. Lucifer yellow transported to the basolateral side was quantified with a luminometer at Ex/Em = 425 nm/350 nm (Fluoroskan Ascent, Helsinki.). Caco-2 cells were treated with MRPs for 1 h prior to exposure to 7.5% ethanol for another 1 h thereafter. Previous work established that 7.5% ethanol was effective at inducing changes in epithelial monolayer resistance without affecting viability [25,26]. Specifically, a Millicell^®^ ERS voltammeter (Millipore Ltd., Etobicoke, Ontario, Canada) was used to measure the electrical resistance between apical and basolateral compartments. TEER values were recorded as Ω.cm^2^. The TEER value was measured both before and after the transport experiments to ensure the integrity of the monolayers. To check for repeatability, all transport experiments were performed at least three times in duplicate assays. Relative TEER values were determined according to Equation (8):

(8) $$\mathrm{Relative}\;\mathrm{epithelial}\;\mathrm{resistance}\;\left(\%\right)=(\;\frac{\mathrm{TEER}\;\mathrm{value}\;\mathrm{for}\;\mathrm{Caco}\text{-}2\;\mathrm{cells}\;\mathrm{after}\;\mathrm{incubation}\;\mathrm{with}\;7.5\%\;\mathrm{ethanol}}{\mathrm{TEER}\;\mathrm{value}\;\mathrm{for}\;\mathrm{Caco}\text{-}2\;\mathrm{cells}\;\mathrm{before}\;\mathrm{incubation}\;\mathrm{with}\;7.5\%\;\mathrm{ethanol}}\;)\times 100$$

##### Visualization of Treated Tight-Junction Proteins

Caco-2 cells (1 × 10^5^/cm^2^) were seeded on 8-well cover slips (Nunc Inc., Naperville, IL, USA) and incubated for three weeks in 0.25 mL MEM, supplemented with 10% FBS (Gibco, Carlsbad, CA, USA) and 100 units/mL of penicillin and 100 μg/mL of streptomycin (Sigma). Twenty-one-day differentiated Caco-2 cells were pre-treated with MRP samples for 60 min, and then sequentially incubated with 7.5% ethanol for another 60 min (scheme shown in Figure 3A). The medium was replaced, and cells were washed three times using ice-cold PBS, fixed in 300 μL of 2% paraformaldehyde for 20 min at room temperature, and then washed again with ice-cold PBS. The Caco-2 cells were treated with 0.1% Triton X-100 for 20 min, washed three times with ice-cold PBS, and treated to remove non-specific binding using 3% bovine serum albumin (BSA) in PBST (PBS + 0.1% Tween 20) as the blocking agent for 60 min. Cells were incubated with primary antibodies that included anti-Claudin 4, anti-occludin, anti-ZO-1, and anti-glyceraldehyde 3-phosphate dehydrogenase (GAPDH) (Abcam Inc., Cambridge, MA, USA) respectively, in blocking buffer at 4 °C in the dark for 60 min [26]. Caco-2 cells were visualized with fluorescein isothiocyanate (FITC)-conjugated Anti-IgG secondary antibody (Abcam Inc., Cambridge, MA, USA) for 60 min. Coverslips were mounted with 0.4% n-propyl gallate in 90% glycerol and sealed. The morphology of tight-junction proteins was observed using a Zeiss fluorescence microscope (Carl Zeiss Microscopy GmbH, Jena, Germany).

##### Western Blotting of Tight-Junction Proteins

Total protein was extracted from Caco-2 cells using lysis buffer, and protein concentrations were adjusted to a known concentration using Bradford reagent (Sigma, St. Louis, MO, USA), before conducting electrophoresis. TJ proteins were resolved on 8% (for occludin and ZO-1) and 15% (for Claudin-4) SDS–polyacrylamide mini-gels using a Mini-PROTEAN^®^ II Multiscreen Apparatus (Bio-Rad Laboratories, Hercules, CA, USA), and then transferred to nitrocellulose membranes (0.2 μm; Bio-Rad). Non-specific binding was ensured using 5% BSA, and membranes were incubated with anti-human primary antibodies, including anti-Claudin 4, anti-occludin, anti-ZO-1, and anti-GAPDH (Sigma, St. Louis, MO, USA) at 4 °C overnight in the dark. Membranes were washed three times with Tris-buffered Saline-Tween (TBST) and incubated with secondary antibody, horseradish peroxidase-conjugated anti-rabbit IgG (Invitrogen, Carlsbad, CA, USA), for 60 min. The membranes were washed three times with TBST, reacted against a Western ECL substrate (Bio-Rad Laboratories (Canada) Ltd., Mississauga, ON, Canada) for 5 min, and analyzed using the ChemiDoc^TM^ MP Imaging System (Bio-Rad Laboratories Ltd., Hercules, CA, USA).

### 2.3. Statistical Analysis

Data were analyzed by one-way ANOVA, followed by Tukey’s HSD test. Correlations determined using SPSS Statistics (v22.0 software, IBM, Armonk, NY, USA). Significant differences were assessed at *p* < 0.05 for differences between treatment means and *p* < 0.01 for correlation analysis.

## 3. Results

### 3.1. Effect of Alphacel on MRP Sugar Recovery and Color

**Figure 1:**
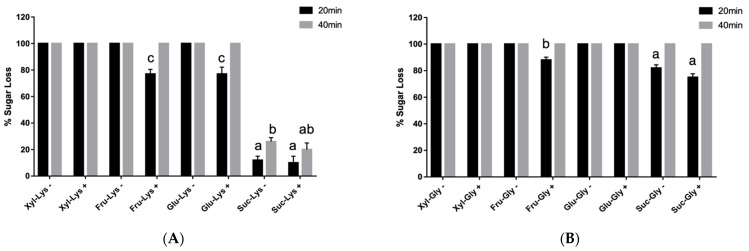
Sugar losses after baking sugar–lysine (**A**) and sugar–glycine (**B**) reactants for 20 and 40 min respectively, at 150 °C. Values represent mean ± SD (*n* = 6). – = without Alphacel; + = with Alphacel. ^a–c^ denote differences in % sugar loss from MRPs derived from different substrates (*p* < 0.05).

Alphacel, a non-reactive fiber, was used to simulate a matrix in simple dry sugar–amino acid mixtures that were heated at 150 °C for 20 and 40 min, respectively. In general, both the presence of Alphacel and the different types of reacting sugar and amino acids in mixtures were factors that influenced the recovery of total soluble MRPs (Appendix A, Table A1). The presence of Alphacel produced relatively small but significant losses (*p* < 0.05) of the reducing sugar fructose, when heated for 20 min with lysine (Figure 1A) or glycine (Figure 1B). Glu-, when present with glycine, was lost at both temperatures. Xylose, on the other hand, was completely lost regardless of the presence of Alphacel and at both heating temperatures for both amino acids, lysine (A) and glycine (B). Fru-glycine and Fru-lysine respectively, when heated in the presence of Alphacel, displayed the lowest (*p* < 0.05) loss of reducing sugar after 20 min of heating. The non-reducing sugar, sucrose, displayed markedly lower (*p* < 0.05) losses compared to the reducing sugar–amino acid products. Proportional losses of sucrose were also relatively lower (*p* < 0.05) when reacted with lysine compared to glycine, when in the presence of Alphacel.

The L*-a*-b* color parameter measurements of water-soluble samples derived from heated dry sugar–amino acid mixtures were used to calculate total color difference (∆E*), a value that refers to the relative degree of browning produced in each model reaction system. The presence of Alphacel in all sugar–amino acid mixtures produced lower (*p* < 0.05) ∆E* values, after 20 min of heating (Table 1). The relative difference in ∆E^*^ value was lower when lysine was the amino acid used in the reaction with reducing sugars, indicating a lower browning rate compared to glycine. All reducing sugar–amino acid mixtures generated higher (*p* < 0.05) ∆E* values than sucrose–amino acid combinations, indicating relatively greater browning attributed to MR than possibly caramelization, which may have been the source of browning in the non-reducing sucrose. There was a positive correlation for the ∆E^*^ value and percentage of sugar lost in reactions with reducing sugars heated for 20 min (*r* = 0.764, *p* < 0.01) and 40 min (*r* = 0.862, *p* < 0.01) respectively, at 150 °C. A similar correlation was not found with sucrose.

### 3.2. MRP Chemical-Based Free-Radical Scavenging Activity

The relative effects of different substrate reactants on different chemical antioxidant activities, when reacted in the presence of Alphacel after 20 min of heating, are presented in Table 2. Extending the heating time to 40 min did not markedly alter the activities (Appendix A, Table A2). All reducing sugar–amino acid model MRPs displayed scavenging activity toward stable DPPH and ABTS^•+^ radicals and AAPH-generated peroxyl radicals, respectively (*p* < 0.05). The greatest scavenging activity was observed for Glu-amino acid mixtures (*p* < 0.05), regardless of whether glycine or lysine was used, albeit the order of antioxidant activity was different between sugars and dependent on the type of amino acid present in some cases. For example, in glycine–sugar models, the relative free-radical scavenging capacity followed a pattern of Glu > Fru ≈ Xyl >> Suc (*p* < 0.05); whereas, when lysine was used, the pattern was Glu ≈ Xyl > Fru >> Suc (*p* < 0.05). The presence of Alphacel in each of the heated reaction mixtures containing reducing sugars decreased (*p* < 0.05) the radical scavenging activity of soluble MRPs, in all chemical assays used herein. In contrast, the non-reducing sucrose displayed relatively very low free-radical scavenging activity in all three assays (*p* < 0.05).

### 3.3. MRP and NO Production in Differentiated Caco-2 Cells

**Figure 2:**
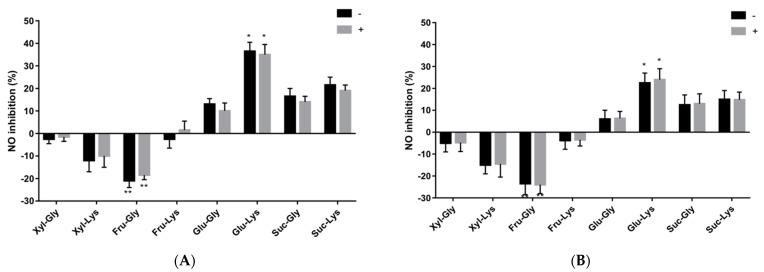
Promotion/inhibition of NO in differentiated Caco-2 cells incubated with sugar–lysine and sugar–glycine. MR reactants for 20 (**A**) and 40 (**B**) minutes respectively, at 150 °C. − = without Alphacel; += with Alphacel. Cells were treated with MRP samples respectively, for 24 h and then stimulated with 8000 U/mL IFN-γ + 0.1 μg/mL PMA for 48 h. Values represent mean ± SD (*n* = 6). * Significant NO inhibition, ** significant NO promotion among different samples (*p* < 0.05).

Soluble products recovered from different sugar–amino acid mixtures heated for 20 min at 150 °C were tested in 21-day differentiated INF-γ + PMA pre-treated Caco-2 cells to determine relative affinity to control NO production (Figure 2A). The type of sugar–amino acid combination used to produce MRPs had an effect on NO production in Caco-2 cells, whereas no effect was observed when Alphacel was present. Heated Glu-lysine MRPs displayed inhibition of NO induction in cells that were relatively greater than the Glu-glycine MRPs (*p* < 0.05). In contrast, Xyl-amino acid and Fru-amino acid mixtures promoted NO synthesis, with relatively greater stimulation (*p* < 0.05) occurring for the Fru-glycine MRP mixture. Extending the heating time to 40 min did not change the relative pattern of MR-induced NO responses in Caco-2 cells, albeit absolute values were slightly increased (Figure 2B). Pre-treating cells with heated non-reducing, sucrose–amino acid mixtures, produced only a small inhibitory effect on INF-γ + PMA-induced NO production.

### 3.4. Effects of MRPs on Caco-2 Cell Viability and Paracellular Permeability

**Figure 3:**
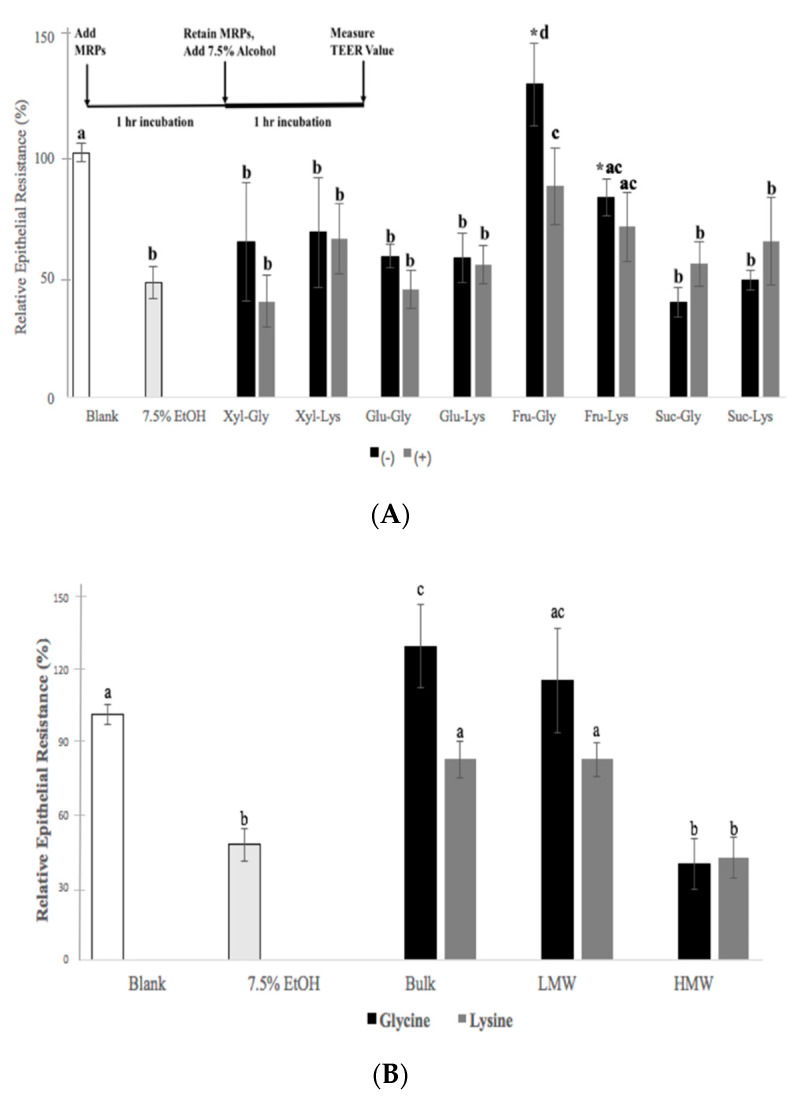
Relative TEER values from Caco-2 cells pretreated with (**A**) different sugar–amino acid MRPs without (−) or with (+) Alphacel and then 7.5% ethanol, values represent mean ± SD (n = 6). (**B**) Fructose–amino acid MRPs (black: Fructose–glycine, grey: Fructose–lysine) without Alphacel and before 7.5% ethanol treatment. Values represent mean ± SD (*n* = 6). Different letters (^a–c^) denote significant differences between treatments (*p* < 0.05). * Denotes differences between Alphacel presence and absence (*p* < 0.05).

The MTT assay was used to assess the viability of Caco-2 cells when exposed to MRPs and alcohol. The MTT assay employs tetrazolium salts to measure the mitochondrial metabolic rate of cultured cells to reduce MTT to an insoluble purple formazan product, and by doing so, indirectly reflects the viable cell numbers. Mitochondria generate reactive oxygen species (ROS) that drive redox-sensitive events and respond to ROS-mediated changes in the cellular redox state. The presence of MRPs at concentrations employed herein were not cytotoxic (MTT values > 90%). Low recovery (5–8%) of Lucifer yellow from the basolateral side of Caco-2 culture also confirmed the integrity of monolayers when exposed to MRPs. The TEER values were also not affected by MRP treatment, confirming the permeability integrity of monolayers. The addition of 7.5% ethanol, however, produced a notable change in cell membrane function, as shown by a significant (*p* < 0.05) reduction in the TEER value from the blank; however, the reduction in TEER corresponded to relatively high MTT values that ranged from 88% to 90%. Pre-treating Caco-2 cells with soluble MRPs produced from 20 min of heating, prior to exposing cells to ethanol, produced large variability on measured TEER values (Figure 3A). In general, only Fru-amino acid mixtures produced significant (*p* < 0.05) compensation for the reduced TEER value observed in cells treated with alcohol, almost approaching initial levels observed with the non-alcohol-treated cells (*p* < 0.05). This was particularly the case with Fru-glycine MRPs, which protected Caco-2 cell TEER values from alcohol exposure to a greater extent than their Fru-lysine counterpart (*p* < 0.05).

Further fractionation of the bulk Fru-glycine-derived MRPs into low and high molecular weight components respectively, revealed that the observed protection from ethanol exposure was attributed only to the low molecular weight fraction (LMW) (Figure 3B). Protection obtained from the LMW fraction was significantly greater (*p* < 0.05) than that obtained from the high molecular weight Fru-glycine fraction and corresponded to what was observed with the bulk MRP.

### 3.5. Effects of MRPs on Caco-2 Cell TEER Values and TJ Membrane Proteins

**Figure 4:**
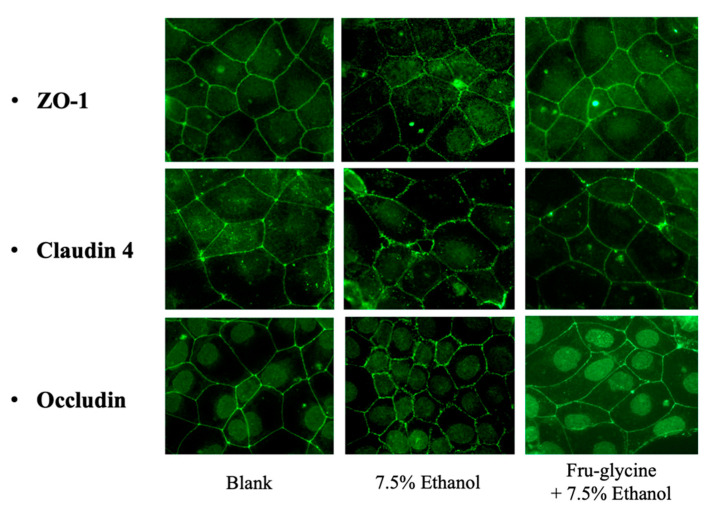
Immunofluorescence staining of Caco-2 TJ proteins, ZO-1, claudin 4, and occludin. Blank = non-treated cells. 7.5% ethanol was the positive control and treatment with Fructose–glycine MRP was prior to ethanol exposure.

**Figure 5:**
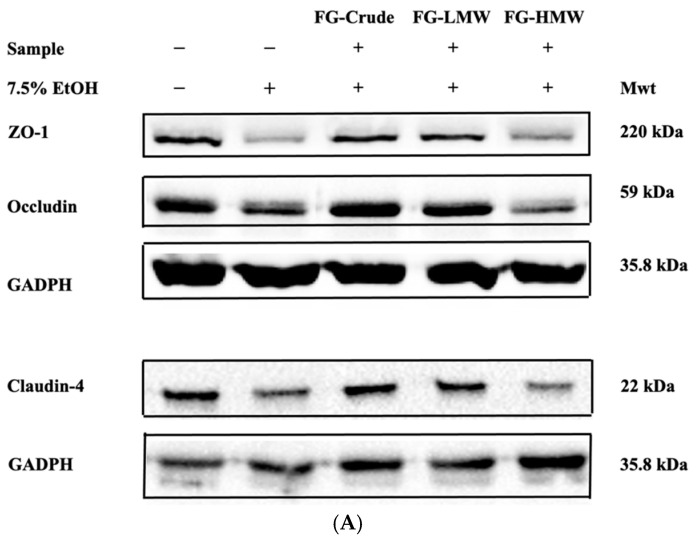

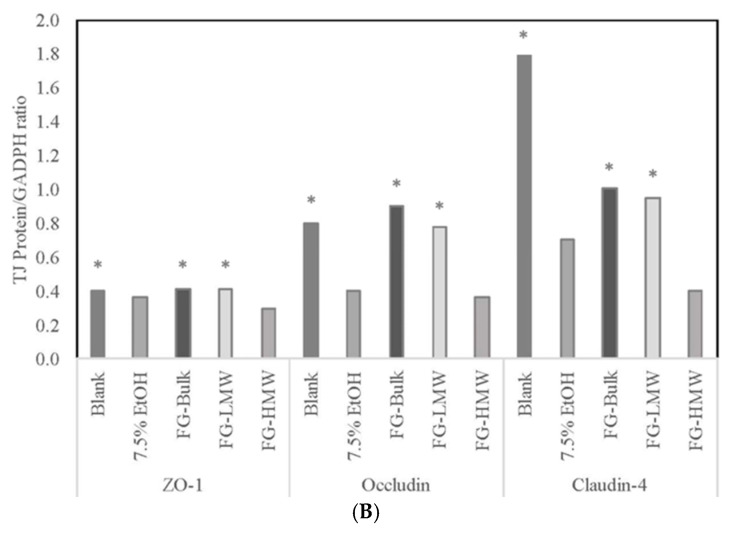
Effect of Fru-glycine (FG) fractions on Caco-2 cell TJ proteins, ZO-1, occludin, and claudin-4 protein expression in ethanol-induced Caco-2 cells. FG-Bulk (crude), FG-LMW (Fru-glycine low molecular weight), FG-HMW (Fru-glycine high molecular weight fractions). (**A**) TJ protein electrophoresis separation with GADPH = glyceraldehyde 3-phosphate dehydrogenase. (**B**) Densitometry readings of separated TJ proteins. Blank (no ethanol), 7.5% ethanol, FG-Bulk (crude), FG-LMW (low molecular weight), FG-HMW (high molecular weight). * Indicates significant (*p* < 0.05) difference between 7.5% ethanol and blank, and FG samples (within groups) respectively, for ZO-1, occludin, and claudin-4, respectively.

The results obtained with cells that were treated with 7.5% alcohol to reduce the intestinal TEER value also produced alterations with integral tight-junction (TJ) membrane proteins that included Claudin 4, occludin, and ZO-1. These proteins are anchored to the actin-based cytoskeleton and are important for regulating paracellular movement of hydrophilic compounds. The Fru-glycine amino acid MRPs which exhibited the greatest relative degree of protection towards intestinal TEER values when exposed to 7.5% alcohol also had visual evidence for an affinity to protect TJ proteins. For example, immunofluorescence staining enabled visualization of Claudin 4, occludin, and ZO-1 proteins, as continuous bands present at intercellular borders in the untreated Caco-2 monolayers (Figure 4). The incubation of cells with 7.5% ethanol dislocated all three TJ proteins from peri-junctional cellular borders, producing visible openings between adjacent cells. Incubating Caco-2 cells with Fru-glycine MRPs prior to alcohol treatment reduced the dislocation of Claudin 4, occludin, and ZO-1, and to some extent, the peri-junctional gap between cells. These findings support the observations made with TEER values on cells exposed to oxidative stress induced by 7.5% ethanol. Confirmation of this finding using electrophoresis to visualize changes in TJ proteins showed that the LMW fraction recovered from Glu-glycine MRP was uniquely active for this protection (Figure 5A). A semi-quantitative analysis of the electrophoresis protein separations confirmed this effect (Figure 5B).

## 4. Discussion

The Maillard reaction represents a complex series of reactions in heated foods that initiate when a carbonyl group from a reducing sugar reacts with an amine from available amino acids or peptides. The kinetics of the reaction are facilitated by heat. Reaction rates as are the distribution of specific products that contribute to flavor (e.g., aromas from early to intermediate stage), texture, and color (e.g., products from late stage) qualities [29,30,31]. In addition, strategies for generating MRPs for food preservation [31,32,33] have been based on reports of antioxidant activity [12,20,34,35]. There has also been interest in the observed health benefits of MRPs attributed to possible carry-over anti-inflammatory effects associated with gut health [22,36,37,38,39]. Due to the chemical complexity of the MR, many studies have relied on using simple aqueous sugar–amino acid model systems to understand the conditions that control the reaction rate and bioactivity of generated products [34,35,38,39,40]. Although great success has been achieved using this approach to further our overall understanding of MR, the absence of a matrix precludes extrapolating results that concern the influence of heat transfer kinetics to produce MRPs in food systems [11]. This study attempted to compare the significance of including a non-reactive matrix along with testing effects of different sugar and amino acid reactants on the quantity and quantity of different model MRPs generated using dry heat. To assess bioactivity, attention was directed at monitoring differences in antioxidant activity using chemical tests and differentiated Caco-2 cells to measure specific intestinal functionalities.

The activation energies for the Maillard reaction activation energy cover a wide range of 23–238 kJ/mol [41], and are dependent on reaction conditions. For example, an activation energy of 170 kJ/mol reported using a sugar–whey protein mixture varies greatly from what is required to bake cookies (e.g., 10.6 kJ/mol) [42]. In the present study, the addition of Alphacel with different reactants appeared to reduce to the rate of the Maillard browning during heating according to observed recovery of soluble browning products, along with relative sugar losses and color changes. The relevance of having Alphacel present during the reaction was specific to the types of sugar and amino acid reactants used to generate MRPs during 20 min of heating. In fact, sugar type was previously shown to contribute relatively more to MR browning than the type of amino acid [21]. Differences in specific melting points for individual sugars [43] and the relative extent of electrophilic carbonyl groups [44] are reasons for the high contribution and specificity of sugars to initiate browning and the production of polymeric late-reaction melanoidin products. In the present study, the addition of Alphacel to different sugar–amino acid mixtures reduced the relative magnitude of sugar losses, particularly for fructose and glucose. The presence of a matrix to either absorb thermal energy, or to hinder energy transfer in the medium uniformly, could explain the notable differences in losses of hexose sugars and the generation of MRPs. Only a small change in sucrose loss and color formation was observed for the non-reducing sucrose–amino acid mixture, regardless of the presence or absence of a matrix. The browning observed with sucrose likely occurred as a result of caramelization attributed to the high heating conditions used, or possibly the initiation of MR after hydrolysis of sucrose to elemental reducing sugars, glucose and fructose, which then reacted with amino acids to generate MRPs.

The chemical antioxidant assays employed herein to assess free-radical scavenging activity of MRPs have been categorized as hydrogen atom transfer (HAT) for the ORAC assay and electron transfer (ET), which describes both the TEAC and DPPH assays, respectively [45]. The presence of Alphacel in sugar–amino acid mixtures produced a small but significant effect on the free-radical scavenging capacities of different MRP products. A temporal pattern of MRP-generated free-radical scavenging properties has been reported previously using similar model systems, but in the absence of a non-reactive matrix, results were attributed to specific early to intermediate MRP products associated with thermolysis of Amadori (aldo)- and Heyns (keto)- (intermediate products [46,47,48]), and also heterocyclic products derived at later stages of the reaction [45,49]. Although the type of reducing sugar has often been the focus for explaining the relative extent of free-radical scavenging, lysine has been regarded as a primary amino acid for this outcome in aqueous model systems [50,51]. Reports have also shown that Glu-lysine MRPs exhibit peroxyl radical scavenging from the chemical ORAC assay, which corresponds to Caco-2 intestinal intercellular antioxidant capacity [5]. It is noteworthy, however, that others observed that glycine produced higher ABTS scavenging activity and melanoidin content, compared to lysine from different bread formulations [2]; and moreover, can accelerate browning when added to a cysteine-xylose reaction [52]. These results suggest that the impact of differences in the food matrix and MR component composition can affect the rate of MR and influence specific products formed, which vary in antioxidant activity. The fact that sucrose displayed markedly lower free-radical scavenging activity regardless of the amino acid type, and the presence of Alphacel, indicates that our findings pertain mainly to non-reducing sugar–amino acid involvement in generating functional MRPs.

The differentiated inflamed Caco-2 enterocyte model used herein characterized the effect of different crude and fractionated MRPs on nitric oxide production [21,22,53]. The model requires pre-exposure of cells to MRP for 24 h to mitigate a subsequent INF-γ + PMA-stimulated inducible nitric oxide synthase (iNOS) response, without interfering with cell viability. Validation of this assay using aminoguanidine and pyrrolidine dithiocarbamate inhibitors of iNOS and monitoring cytokine responses in Caco-2 cells has been reported [22]. In the present study, the presence of Alphacel along with different sugar–amino acids to generate different MRPs within 20 min of heating produced no differences in MRP-induced Caco-2 cell NO production. Extending the heating time of the same reactants to 40 min did not dramatically change the magnitude of MRP-induced effects on NO production. Moreover, regardless of the presence of Alphacel, or difference in heating time in this study to generate MPRs’ production, the type of reducing sugar was the primary factor for evoking a simultaneous effect on NO production in cytokine-treated cells. A lesser effect was observed using lysine or glycine respectively, to generate different MRPs. Inhibiting the IFN-γ-PMA cytokine induced NO production in Caco-2 cells with Glu-lysine- and Glu-glycine-generated MRPs pre-treatment, which confirmed the results of our former study, that used similar baking temperature and times to generate bioactive MRPs [21]. However, in direct contrast was the observation that MRPs derived from heated Fru-amino acid mixtures synergistically stimulated NO production in cytokine-treated Caco-2 cells. A lesser stimulation was also observed with the Xyl-lysine MRP. Advanced glycation end-products produced in other studies demonstrated activity to activate nuclear factor Kappa-B in macrophages and induce NO release [54]. Hence, the type of sugar and amino acid used to generate MRPs should be particularly relevant for generating NO production in inflamed Caco-2 cells. In the present study, enhanced stimulation of NO in INF-γ + PMA-induced Caco-2 cells, that were pretreated with Fru-glycine MRPs, did not result in Caco-2 cell cytotoxicity, thus NO concentrations reached by stimulation were not toxic to the cells.

The activity of NO to induce or abate dysfunction of intestinal cell barrier and function depends on the cellular concentration. At low levels, NO can mitigate oxidative stress by reducing free-radical and metal-catalyzed lipid peroxidation, and serve to trigger signal transduction mechanisms that control gene expression of antioxidant enzymes [55,56]. Alternatively, excess NO production can lead to generation of reactive peroxynitrate radicals, which degrade cytoskeletal proteins, leading to epithelial dysfunction. Using the Caco-2 cell model to monitor changes on the intestinal epithelial barrier [24,26], the pre-treatment with MRPs derived only from Fru-glycine MRPs conferred a significant protective effect when the epithelial cells are subjected to acute alcohol treatment. Ethoxy radicals produced from alcohol evoke an oxidation and reduction imbalance that disrupts barrier function in Caco-2 epithelial cells [23]. Recent studies have reviewed the important capacity of bioactive food components, such as polyphenols and probiotic fermentation products, to maintain, or increase, TEER in order to protect against a loss in intestinal permeability [57,58]. The capacity for Fru-glycine MRPs to protect against alcohol-induced lowering of TEER values and reduce disruption of the cytoskeleton (e.g., expression of tight-junction protein) was shown for the first time in this study. Taken together with the free-radical scavenging activity and affinity to synergistically stimulate NO in cytokine-treated intestinal cells, MRPs derived specifically from Fru-glycine reactants were shown to have a potential important role to protect epithelial function from oxidative stress, which was not observed previously by other bioactive MRPs.

A common outcome of many different in vitro cell-based model studies, that focused on numerous bioactivities observed for both crude Glu- and Fru- MRP mixtures, identified a small molecular weight component(s) that elicited activity [21,50,59,60,61]. A similar observation was made herein, where a LMW (e.g., <1 kDa) fraction recovered from the bulk Fru-glycine MRP was attributed to the protection observed in the TEER value and TJ protein dislocation, induced by alcohol in differentiated Caco-2 cells. Albeit challenging, further studies are required to attempt to isolate and characterize this and possibly other individual active components of Fru-glycine MRPs that display this capacity.

## 5. Conclusions

This study adds to our understanding on how generation of MRPs can vary depending on the presence of a definable matrix, and moreover, the different types of reactant sugars and amino acids used as precursors in the reaction. The inclusion of Alphacel as a non-reactive matrix contributed only to a small reduction in MRP yield, but produced significant changes in antioxidant activities, thereby suggesting that either absorbance or hindrance of energy transfer attributed to a matrix can to some extent influence the quality of MRPs recovered under conditions used in this study. Future studies will be necessary to determine to what extent Alphacel changed the composition of different MRP products when present during heating. Varying the type of reducing sugar produced the greatest influence on both yield and specific functionality of MRPs formed by dry heat. A LMW component recovered from crude Fru-glycine MRP produced unique protection for Caco-2 cells’ TEER value, and was for the first time shown to be related to a capacity to stabilize TJ proteins under conditions of acute oxidative stress. This is another example of a specific bioactivity for LMW components isolated previously from crude MRPs. The next challenge in MR research will be to isolate and characterize additional LMW components of MRPs that have relevance to food sources commonly consumed and which may also contribute directly to health gut.

## Figures and Tables

**Table 1:** Color change parameter, ∆E*, of water-soluble samples derived from sugar–amino acid heated with and without 10% Alphacel in model MR systems ^1^.

Sample		20 min		40 min	
		(−)	(+)	(−)	(+)
Glycine	Xylose	81.27 ± 0.87 ^ex^	76.69 ± 0.41 ^dy^	80.44 ± 0.21 ^cx^	80.29 ± 0.07 ^cx^
	Fructose	79.87 ± 0.11 ^dx^	73.23 ± 0.01 ^dy^	80.71 ± 0.43 ^cx^	81.64 ± 0.33 ^cx^
	Glucose	83.23 ± 0.50 ^ex^	78.17 ± 0.78 ^ey^	75.15 ± 1.48 ^cx^	81.07 ± 1.61 ^cx^
	Sucrose	27.89 ± 0.33 ^ax^	18.54 ± 0.35 ^ay^	74.03 ± 0.81 ^cx^	66.85 ± 0.98 ^cx^
Lysine	Xylose	75.00 ± 0.49 ^dx^	71.76 ± 1.36 ^dy^	73.36 ± 0.34 ^cx^	74.98 ± 1.56 ^cx^
	Fructose	55.39 ± 0.30 ^cx^	46.36 ± 0.18 ^cy^	74.55 ± 1.10 ^cx^	69.24 ± 2.77 ^cx^
	Glucose	74.04 ± 1.16 ^dx^	58.39 ± 1.01 ^cy^	66.45 ± 1.21 ^bx^	65.34 ± 0.29 ^bx^
	Sucrose	42.96 ± 2.52 ^bx^	23.26 ± 0.37 ^by^	47.47 ± 0.12 ^ax^	46.14 ± 0.30 ^ax^

^1^ MR = Maillard Reaction. Heated at 150 °C for 20 and 40 min. Values represent mean ± SD (*n* = 6). (−), (+) represent sugar–amino acid model systems without (−) and with (+) 10% Alphacel, respectively. Superscripts ^a–d^ in columns denote significant difference (*p* < 0.05) in reactants. ^x,y^ in rows denote significant difference (*p* < 0.05) with the presence of Alphacel matrix.

**Table 2:** Antioxidant activities of 20 min baked sugar–amino acid model systems ^1^.

Samples		Assays ^2^					
		DPPH (% Inhibition)		TEAC (mmol TE Per g Sample)		ORAC (µmol TE Per g Sample)	
		(−) ^3^	(+)	(−)	(+)	(−)	(+)
Glycine	Xylose	43.20 ± 1.80 ^cx^	34.61 ± 2.22 ^by^	0.39 ± 0.02 ^bx^	0.27 ± 0.03 ^by^	304 ± 28 ^bx^	275 ± 29 ^by^
	Fructose	47.89 ± 2.64 ^cx^	38.13 ± 2.18 ^cy^	0.35 ± 0.02 ^bx^	0.27 ± 0.01 ^by^	510 ± 27 ^cx^	412 ± 20 ^cy^
	Glucose	57.76 ± 3.32 ^dx^	43.12 ± 2.71 ^dy^	0.48 ± 0.02 ^cx^	0.36 ± 0.03 ^cy^	562 ± 29 ^cx^	439 ± 28 ^cy^
	Sucrose	4.83 ± 0.31 ^ax^	4.96 ± 0.84 ^ax^	0.01 ± 0.00 ^ax^	0.01 ± 0.00 ^ax^	4.60 ± 0.98 ^ax^	4.5 ± 1.2 ^ax^
Lysine	Xylose	48.90 ± 3.39 ^cx^	36.60 ± 2.03 ^cy^	0.37 ± 0.01 ^bx^	0.23 ± 0.01 ^by^	382 ± 26 ^bx^	309 ± 18 ^by^
	Fructose	38.68 ± 1.19 ^bx^	27.12 ± 1.11 ^by^	0.28 ± 0.02 ^bx^	0.19 ± 0.01 ^by^	365 ± 23 ^bx^	277 ± 23 ^by^
	Glucose	50.56 ± 3.07 ^dx^	38.31 ± 2.62 ^cy^	0.39 ± 0.01 ^bx^	0.30 ± 0.01 ^bx^	416 ± 37 ^cx^	335 ± 42 ^by^
	Sucrose	5.83 ± 0.52 ^ax^	5.72 ± 0.39 ^ax^	0.02 ± 0.00 ^ax^	0.02 ± 0.00 ^ax^	13.8 ± 3.2 ^ax^	14.9 ± 2.7 ^ax^

^1^ Value represents mean ± SD (*n* = 3), significant differences were analyzed with ANOVA with Tukey’s HSD test. Superscripts ^a–c^ denote significant differences (*p* < 0.05) in columns. ^x,y^ denote significant differences (*p* < 0.05) in rows. Samples heated at 150 °C for 20 min. ^2^ DPPH represents DPPH radical scavenging antioxidant capacity, values are expressed as % inhibition. TEAC represents Trolox equivalent antioxidant capacity, values expressed as mmol Trolox equivalents (TE)/g samples. ORAC represents oxygen radical absorption capacity, values expressed as µmol TE per g samples. ^3^ (−) without Alphacel matrix, (+) with Alphacel matrix.

## Data Availability

The data is contained within the article.

## Abbreviations

ABTS, 2,2′-Azino-bis-(3-ethylbenzothiazoneline-6-sulfonic acid); AAPH, 2,2′-azobis(2-amidino-propane) dihydrochloride; DPPH, 2,2-diphenyl-1-picrylhydrazyl; FBS, fetal bovine serum; FITC, fluorescein isothiocyanate; Fru-glycine, fructose-glycine; Fru-lys, fructose-lysine; Glu-glycine, glucose-glycine; HMW, high molecular weight Maillard reaction products; GAPDH, glyceraldehyde 3-phosphate dehydrogenase; LMW, low molecular weight Maillard reaction products; MTT, 3-(4,5-dimethylthiazol-2-yl)-2,5-diphenyltetrazolium bromide; MR, Maillard reaction; NO, nitric oxide; ORAC, oxygen radical absorbance capacity; PBS, phosphate-buffered saline; TEAC, Trolox equivalent antioxidant capacity; TEER, transepithelial electrical resistance; TJ, tight junction; Xyl-glycine, xylose-glycine; Xyl-lysine, xylose-lysine; ZO-1, zonula occluden-1.

## Appendix A

**Table A1:** Yield (%*w/w*) of water-soluble reactants following sugar–amino acid heated with and without 10% Alphacel in model MR systems ^1^.

Sample		20 min		40 min	
		(+)	(−)	(+)	(−)
Glycine	Xylose	49.48 ± 0.06 ^bx^	47.30 ± 0.02 ^bx^	36.23 ± 0.06 ^bx^	36.12 ± 0.02 ^bx^
	Fructose	38.53 ± 0.05 ^ax^	33.53 ± 0.09 ^ay^	27.67 ± 0.0 ^ax^	22.57 ± 0.07^ay^
	Glucose	65.73 ± 0.08 ^dx^	55.52 ± 0.05 ^cy^	45.81 ± 0.01 ^cx^	43.02 ± 0.04 ^cy^
	Sucrose	86.78 ± 0.08 ^cx^	82.05 ± 0.07 ^dy^	86.47 ± 0.08 ^dx^	80.87 ± 0.07 ^dy^
Lysine	Xylose	41.89 ± 0.03 ^ax^	42.65 ± 0.05 ^ax^	33.64 ± 0.02 ^ax^	35.29 ± 0.12 ^ax^
	Fructose	43.76 ± 0.03 ^ax^	40.44 ± 0.04 ^ax^	40.08 ± 0.02 ^ax^	37.30 ± 0.03 ^ay^
	Glucose	77.21 ± 0.18 ^bx^	70.64 ± 0.0 ^by^	73.97 ± 0.02 ^bx^	70.34 ± 0.02 ^by^
	Sucrose	98.63 ± 0.06 ^cx^	76.48 ± 0.02 ^by^	98.52 ± 0.06 ^cx^	76.25 ± 0.02 ^by^

^1^ MR: Maillard Reaction, heated at 150 °C. Values represent mean ± SD (*n* = 6). (+), (−) represent sugar–amino acid model systems without (−) and with (+) 10% Alphacel, respectively. Samples were heated for 20 and 40 min at 150 °C. Yield refers to the presence of both unreacted solutes and generation of water-soluble MRP. ^a–d^ Means in columns with different superscript letters denote significant differences (*p* < 0.05) in reactants. ^x,y^ Means in rows with different letters denote significant differences (*p* < 0.05) with the presence of Alphacel matrix.

**Table A2:** Antioxidant activities of baked sugar–amino acid model systems ^1^.

Samples		Assays ^2^					
		DPPH (% Inhibition)		TEAC (mg/mL Trolox Equivalent)		ORAC (µmol/Trolox Per g Samples)	
		(−) ^3^	(+)	(−)	(+)	(−)	(+)
Glycine	Xylose	36.76 ± 7.41 ^bx^	35.41 ± 8.23 ^bx^	0.33 ± 0.03 ^bx^	0.32 ± 0.01 ^bx^	369 ± 23 ^bx^	273 ± 26 ^by^
	Fructose	44.34 ± 5.23 ^cx^	44.21 ± 3.78 ^cx^	0.40 ± 0.02 ^bx^	0.33 ± 0.0 ^bx^	512 ± 28 ^cx^	434 ± 21 ^cy^
	Glucose	27.45 ± 2.62 ^bx^	26.41 ± 2.91 ^bx^	0.39 ± 0.02 ^bx^	0.37 ± 0.03 ^bx^	555 ± 26 ^cx^	424 ± 28 ^cy^
	Sucrose	4.32 ± 0.81 ^ax^	4.27 ± 0.65 ^ax^	0.02 ± 0.00 ^ax^	0.01 ± 0.0 ^ax^	5.44 ± 0.9 ^ax^	4.83 ± 1.2 ^ax^
Lysine	Xylose	27.53 ± 4.19 ^bx^	26.75 ± 3.68 ^bx^	0.23 ± 0.01 ^bx^	0.23 ± 0.01 ^by^	377 ± 27 ^bx^	355 ± 16 ^bx^
	Fructose	39.36 ± 6.29 ^c^	38.74 ± 4.89 ^c^	0.40 ± 0.02 ^bx^	0.193 ± 0.0 ^by^	354 ± 27 ^bx^	296 ± 29 ^bx^
	Glucose	24.31 ± 3.67 ^bx^	23.73 ± 3.22 ^bx^	0.35 ± 0.01 ^bx^	0.31 ± 0.0 ^bx^	419 ± 34 ^bx^	355 ± 38 ^by^
	Sucrose	5.76 ± 0.42 ^ax^	5.31 ± 0.41 ^ax^	0.03 ± 0.0 ^ax^	0.02 ± 0.0 ^ax^	12.8 ± 3.3 ^ax^	14.0 ± 2.9 ^ax^

^1^ Value represents mean ± SD (*n* = 3), significant differences were analyzed with ANOVA with Tukey’s HSD test. Superscripts ^a–c^ denote significant differences (*p* < 0.05) in columns. ^x,y^ Denote significant differences (*p* < 0.05) in rows. Samples heated for 40 min at 150 °C. ^2^ DPPH represents DPPH radical scavenging antioxidant capacity, values are expressed as % inhibition. TEAC represents Trolox equivalent antioxidant capacity, values expressed as mg/mL Trolox equivalents/g samples. ORAC represents oxygen radical absorption capacity, values expressed as µmol/Trolox per g samples. ^3^ (−) without Alphacel matrix, (+) with Alphacel matrix.
